# Manual Therapy as an Alternative Treatment Option for Idiopathic Pulmonary Fibrosis: A Case Report

**DOI:** 10.7759/cureus.53383

**Published:** 2024-02-01

**Authors:** Sally S Greenberg, Sydney E Moriarty, Ishan Perera, Hannah E Kasper, Bradley Kasper, Holly Moriarty

**Affiliations:** 1 Medical School, Edward Via College of Osteopathic Medicine, Blacksburg, USA; 2 Sports Chiropractic, Haymarket Chiropractic and Rehabilitation, Gainesville, USA

**Keywords:** active release technique, pulmonary function test, manual therapy, interstitial lung disease, idiopathic pulmonary fibrosis

## Abstract

Idiopathic pulmonary fibrosis (IPF) is a type of interstitial lung disease that results in fibrotic lung tissue leading to symptoms of dyspnea, nonproductive cough, decreased mobility, and fatigue. This case report presents a well-documented case of a 73-year-old female presenting to a chiropractic and rehabilitation center seeking care after failing medication therapy. The patient presented hypoxic on room air with decreased mobility throughout her cervical, thoracic, and lumbar spine. Three manual therapy techniques were performed at the rehabilitation clinic aimed at improving the range of motion of the impacted joints and surrounding tissue. The patient was also given manual therapy exercises to perform at home. Pre- and post-intervention assessments showed an improvement in oxygenation saturation on room air, increased mobility, and stabilization of the patient's pulmonary function test (PFT) values. This case demonstrates the importance of considering manual therapy in patients who have failed the standard of care or are unable to tolerate the medications used to treat IPF.

## Introduction

Idiopathic pulmonary fibrosis (IPF) is a lung disorder of unknown cause that leads to progressive scarring of the lungs, hardening of the tissue, and a resulting decreased pulmonary function [1-2]. Patients with exposure to wood and metal dust, livestock, microaspirations, or tobacco smoke have an increased risk of IPF [3-4]. IPF commonly presents with dyspnea, a nonproductive cough, decreased mobility, and fatigue [1-2]. Disease progression is variable for each patient, with a median survival rate of less than five years after a patient is diagnosed with IPF [2,5]. There is currently no cure for IPF, with treatment focusing on slowing the progression of the disease while stabilizing the patient [6]. The current standard of care includes two medications, nintedanib and pirfenidone, along with oxygen therapy [1-2,6-7]. If a patient fails standard therapy, or standard therapy alone is insufficient, pulmonary rehabilitation may be added with consideration of lung transplantation [1,7]. Here, we present a case detailing how pulmonary rehabilitation, in the form of manual therapy, improved a patient's oxygenation and mobility with stabilization of her pulmonary function test (PFT) values.

## Case presentation

History and examination

A 73-year-old female with a past medical history of IPF and gastroesophageal reflux disease (GERD) presented to a chiropractic and rehabilitation center looking for pulmonary rehabilitation after failing standard medication therapy for IPF. The patient was intolerant to both pirfenidone, due to the negative side effects experienced, and nintedanib, due to the elevation of her liver function tests (LFTs) following medication treatment. Upon presentation, the patient complained of dyspnea with a modified Medical Research Council (mMRC) grade of 4 and decreased mobility. A detailed physical exam including evaluation of range of motion, palpation, and special tests was performed revealing multiple joint restrictions throughout the spine resulting in hypomobility, spasms, and endpoint tenderness in her cervical, thoracic, and lumbar spine. It was also noted that the patient had decreased rib expansion during ventilation.

Rehabilitation plan

**Figure 1:**
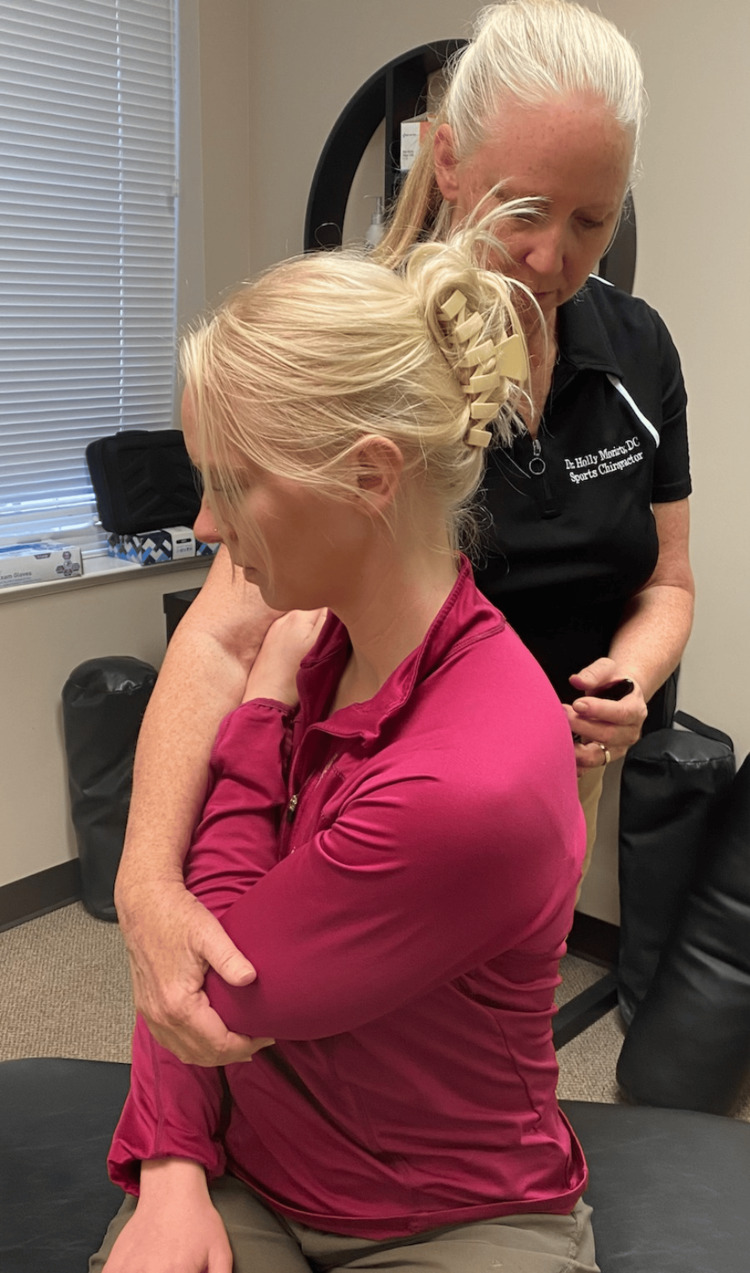
Anterior view of joint mobilization with activator Participants pictured in the figure are researchers in the study, not the patient

**Figure 2:**
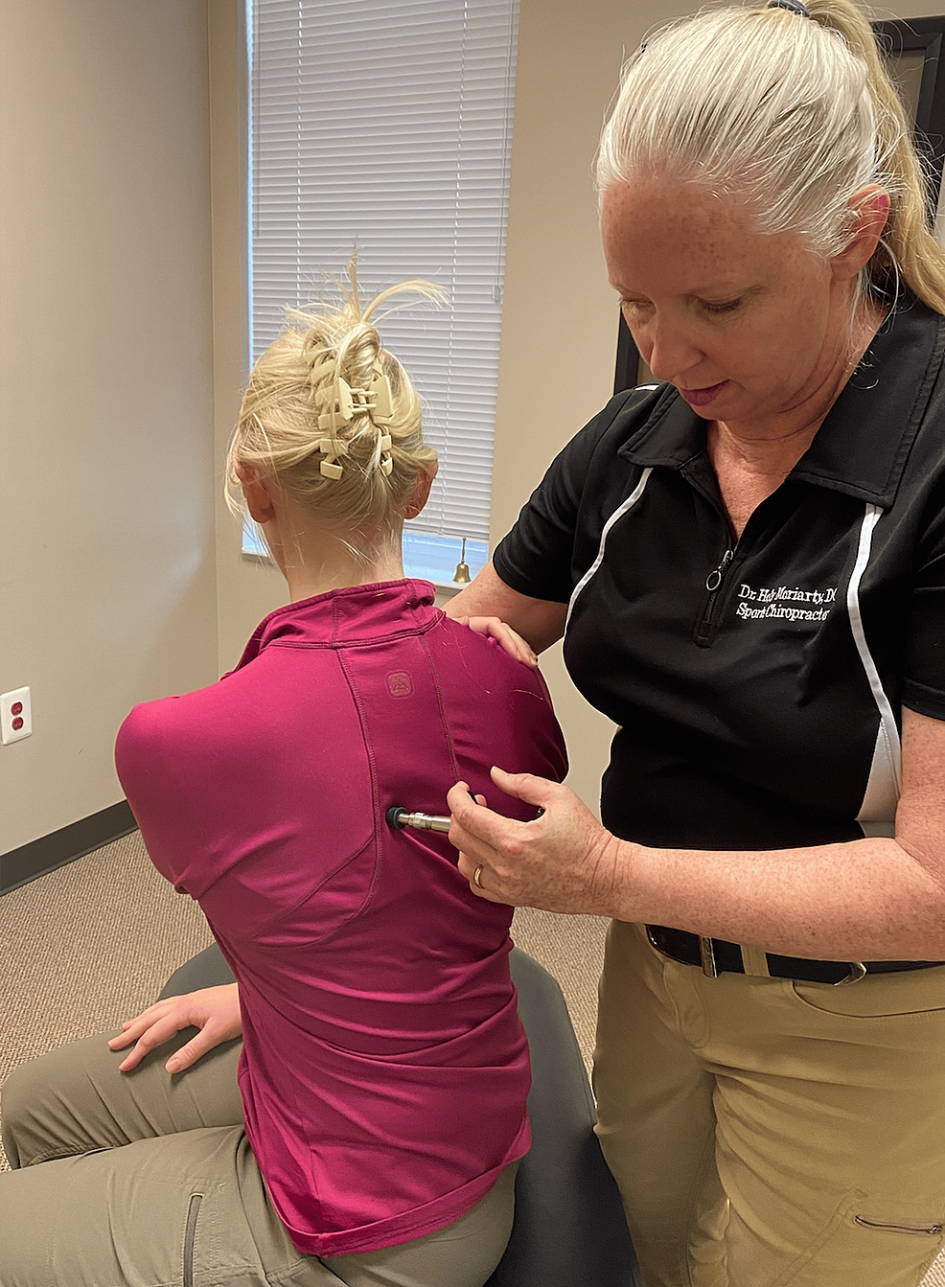
Posterior view of joint mobilization with activator Participants pictured in the figure are researchers in the study, not the patient

**Figure 3:**
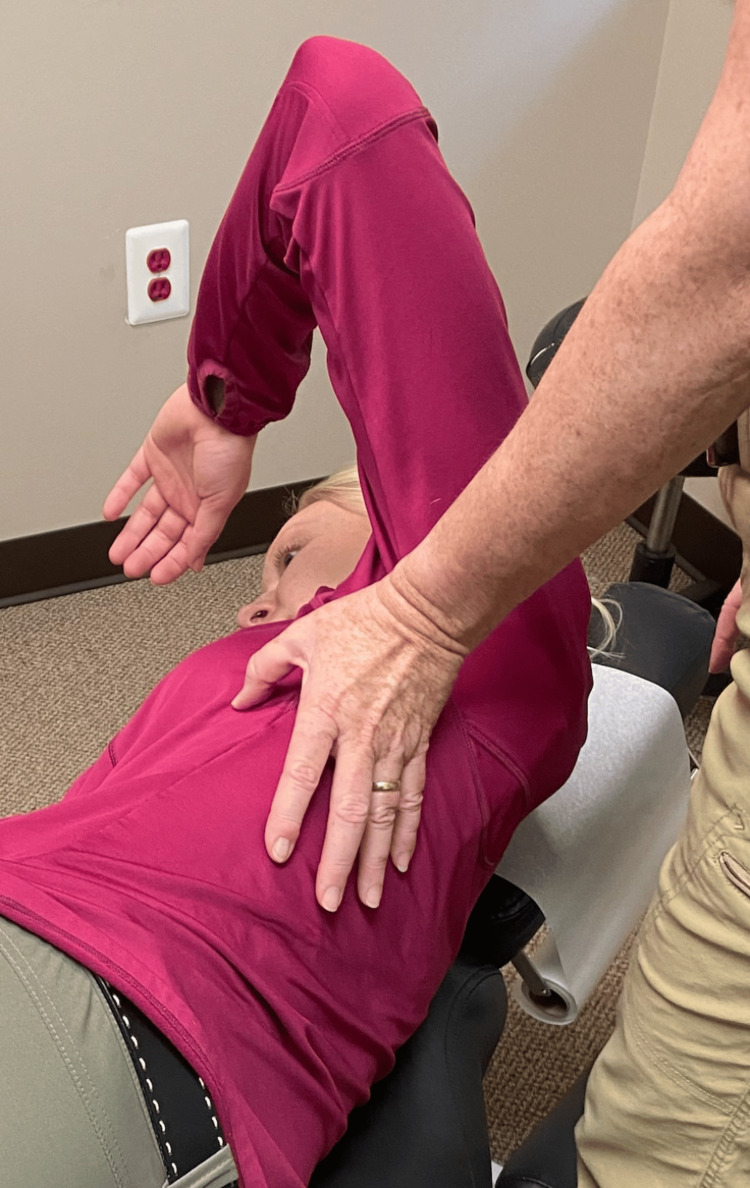
MRT on intercostal muscles Participants pictured in the figure are researchers in the study, not the patient MRT: myofascial release technique

**Figure 4:**
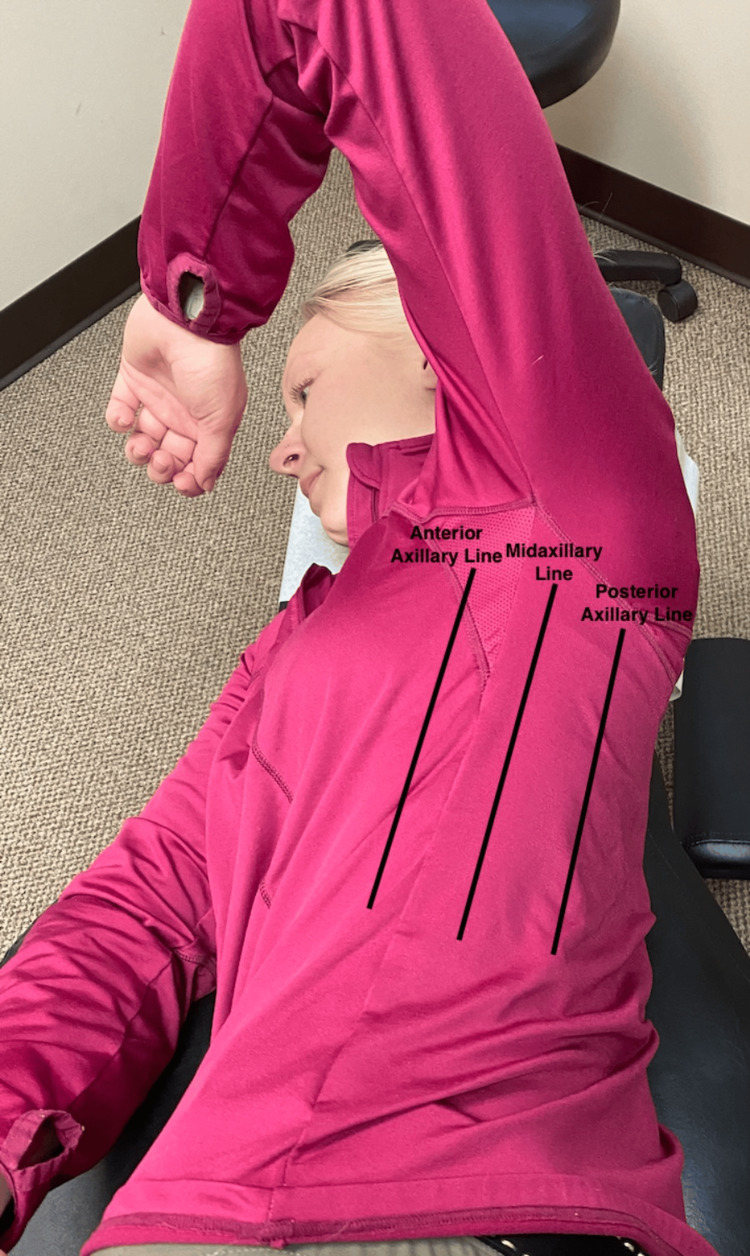
Labeled image of axillary lines used in MRT on intercostal muscles The participant pictured in the figure is a researcher in the study, not the patient MRT: myofascial release technique

**Figure 5:**
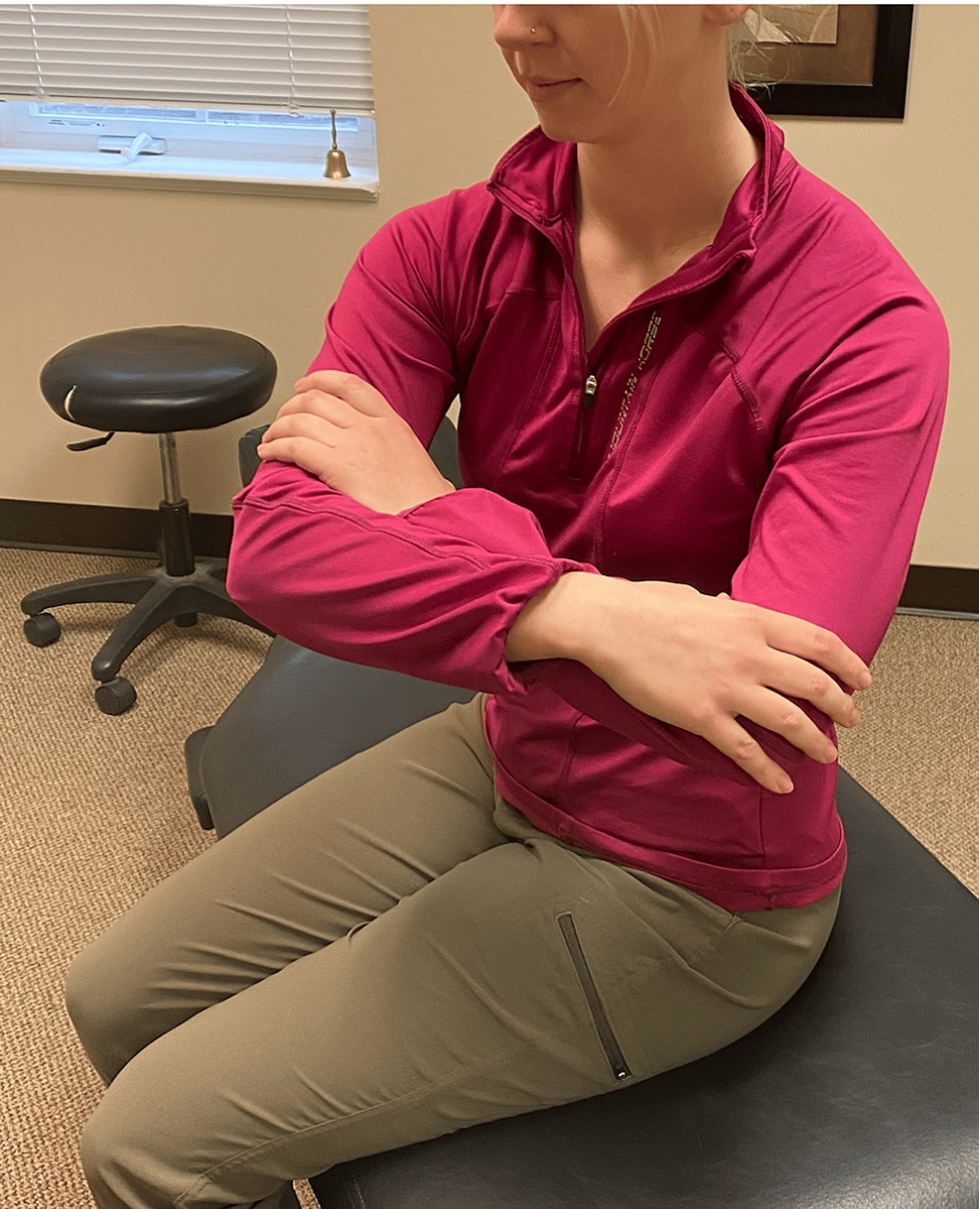
Initial position of trunk rotation exercise The participant pictured in the figure is a researcher in the study, not the patient

**Figure 6:**
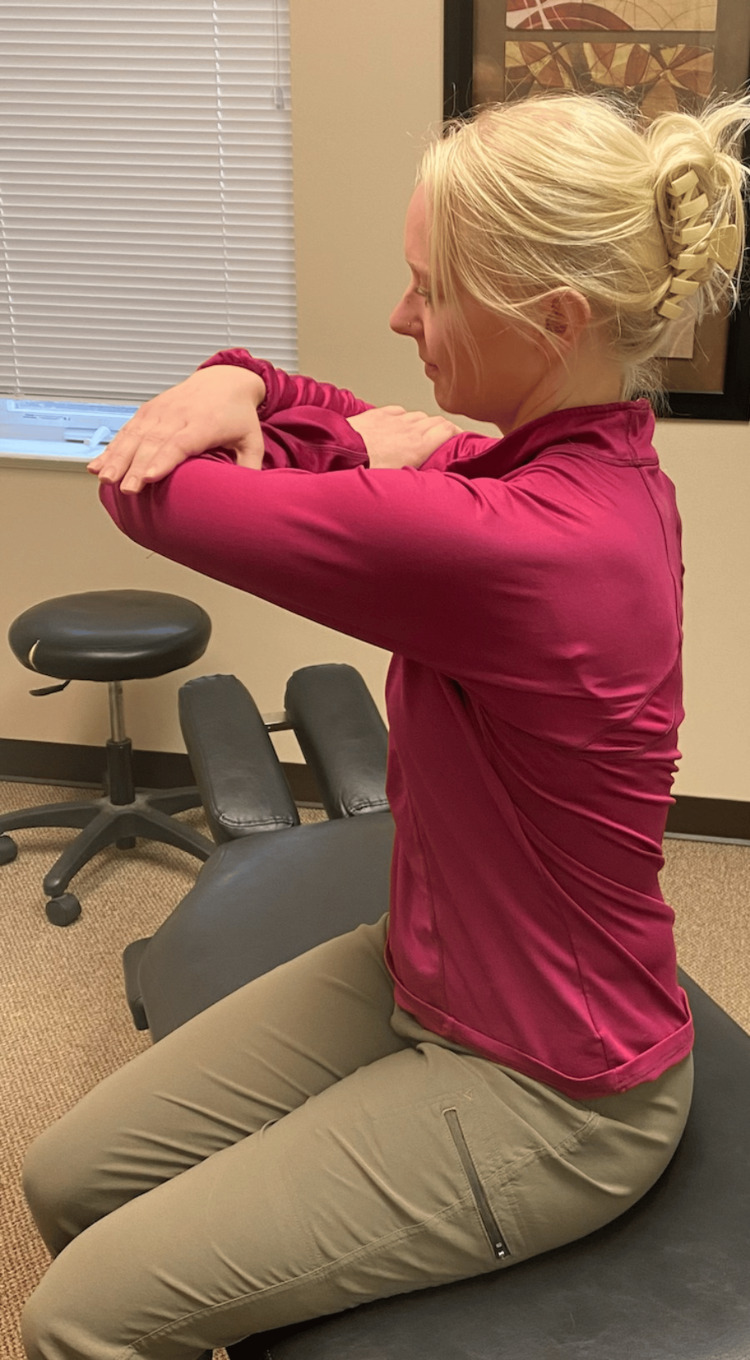
Trunk rotation exercise The participant pictured in the figure is a researcher in the study, not the patient

**Figure 7:**
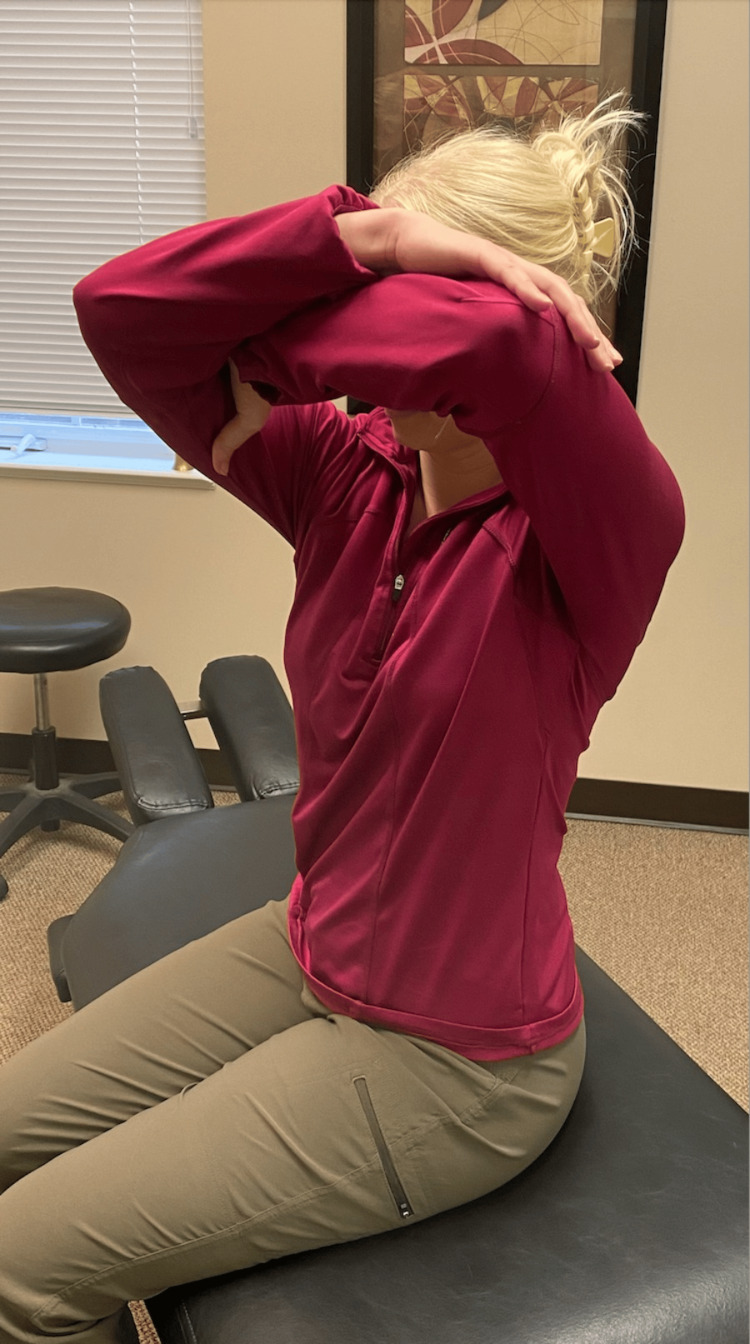
Final position of trunk rotation exercise The participant pictured in the figure is a researcher in the study, not the patient

Following a review of the patient's records and history of present illness, and discussing treatment options, it was determined that the best treatment for the patient's symptoms included the following three manual therapy techniques: joint mobilization with an activator, intercostal muscle stretching with usage of myofascial release technique (MRT), and trunk rotation with arm elevation. The goal of the joint mobilization with an activator was to align the joints, increase the mobility of the spine, and allow for improved ventilation. The technique was conducted in the seated position, for patient comfort, and required the patient to twist in one direction while an activator was used targeting the transverse process, with the inclusion of the costovertebral joints when in the thoracic spine, aimed 15-20 degrees laterally (Figures 1, 2). This was then repeated bilaterally in both directions on her cervical, thoracic, and lumbar spine. The second technique was MRT on the intercostal muscles (Figure 3). The patient laid in the left lateral recumbent position to perform MRT on the right intercostals and then in the right lateral recumbent position to perform MRT on the left intercostals. MRT was performed bilaterally along the anterior axillary line, midaxillary line, and posterior axillary line (Figure 4). While the clinician was performing the MRT, the patient’s arm was in an abducted position with flexion at the elbow to allow for comfort. The third technique was trunk rotation with arm elevation. This technique aimed to stretch the intercostal muscles, allowing for improved rib expansion as felt and reported by the patient (Figures 5, 6, 7). The patient was advised to perform trunk rotation with arm elevation exercises at home daily while also receiving treatment at the outpatient clinic one to three times per week for a total of 10 weeks. Patient symptoms and presentation at each visit decided the frequency of treatment and the addition of alternative techniques. The patient reported that she was compliant with home exercises.

Outcome

Prior to initial treatment, while at the outpatient clinic, the patient's oxygen saturation was fluctuating around 85% on room air. The patient reported she was experiencing dyspnea consistently, which increased upon exertion. Additionally, the patient reported she had a frequent cough, had decreased mobility, and required supplemental oxygen during the day. Following one therapy session, the patient’s oxygen saturation increased to 90% on room air and remained steady without fluctuation. Following multiple sessions, the patient reported she was able to do things she was not able to prior, including walking to and from her barn to brush her horses without dyspnea, with an mMRC grade decrease from 4 to 2. She also reported increased mobility since treatments began, less coughing, and no longer requiring supplemental oxygen during the day. Overall, the patient expressed that she experienced a decrease in her overall symptoms and a positive change in her lifestyle since beginning treatments that remained stable throughout the duration of care. In addition, the patient’s PFT values had stabilized as reported by her pulmonologist (Table 1).

**Table 1:** Pre-therapy PFTs vs. post-therapy PFTs. Pre-therapy data represents PFTs from February. Post-therapy data represents PFTs from May of the same year FVC: forced vital capacity; FEV1: forced expiratory volume in one second; FEF: forced expiratory flow; FIF: forced inspiratory flow; DLCOunc: diffusing capacity of the lungs for carbon monoxide uncorrected; DLCOcor: diffusing capacity of the lungs for carbon monoxide corrected; DL/VA: diffusing capacity of the lungs for carbon monoxide divided by the alveolar volume; VA: alveolar volume; IVC: inspiratory vital capacity; SVC: slow vital capacity; TLC: total lung capacity; RV: residual volume; TGV: thoracic gas volume; ERV: expiratory reserve volume; Raw: airway resistance; sGaw: specific airway conductance; LLN: lower limit of normal; ULN: upper limit of normal

Pre therapy						Post therapy					
Spirometry	Pred	Actual	LLN	ULN	% Pred	Spirometry	Pred	Actual	LLN	ULN	% Pred
FVC (L)	2.89	2.05	2.09	3.72	70	FVC (L)	2.85	2.06	2.05	3.69	72
FEV1 (L)	2.22	1.73	1.61	2.82	77	FEV1 (L)	2.19	1.79	1.58	2.78	81
FEV1/FVC (%)	78	85	64	89	108	FEV1/FVC (%)	78	87	64	89	111
FEF 25-75% (L/sec)	1.84	2.29	0.83	3.29	124	FEF 25-75% (L/sec)	1.81	2.45	0.81	3.25	135
FEF max (L/sec)	5.65	6.67	3.88	7.42	118	FEF max (L/sec)	3.57	7.86	3.8	7.34	141
Expiratory time (sec)		7.1				Expiratory time (sec)		7.16			
FEF 50% (L/sec)	3.28	4.22	1.47	5.1	128	FEF 50% (L/sec)	3.25	3.68	1.44	5.07	113
FIF 50% (L/sec)	3.31	2.13	1.87	4.74	64	FIF 50% (L/sec)	3.25	4.15	1.81	4.68	127
FEF 50%/FIF 50% (%)	90-100	198				FEF 50%/FIF 50% (%)	90-100	89			
Diffusion						Diffusion					
DLCOunc (ml/min/mmHg)	19.98	11.09	14.15	29.25	55	DLCOunc (ml/min/mmHg)	19.91	13.67	14.08	29.2	68
DLCOcor (ml/min/mmHg)	19.98		14.15	29.25		DLCOcor (ml/min/mmHg)	19.91		14.08	29.2	
DL/VA (ml/min/mmHg)	3.84	3.11			80	DL/VA (ml/min/mmHg)	3.83	3.86			100
VA (L)	5.2	3.57	4.32	6.09	68	VA (L)	5.2	3.55	4.32	6.09	68
IVC (L)		1.97				IVC (L)		1.86			
Lung volumes						Lung volumes					
SVC (L)	2.89	2.11	2.09	3.72	73	SVC (L)	2.85	1.87	2.05	3.69	65
TLC (pleth)(L)	5.2	3.45	4.13	6.28	66	TLC (pleth)(L)	5.2	3.86	4.13	6.28	74
RV (pleth)(L)	2.28	1.34	1.52	3.04	58	RV (pleth)(L)	2.3	1.99	1.54	3.06	86
RV/TLC (pleth)(%)	44	39	33	55	87	RV/TLC (pleth)(%)	45	52	34	56	115
TGV (L)	2.98	2.51	1.94	4.03	84	TGV (L)	2.99	2.62	1.94	4.03	87
ERV (L)	1.01	1.18			116	ERV (L)	0.99	0.63			63
Raw (cmH2O/L/s)	1.86	1.06	1.15	2.56	57	Raw (cmH2O/L/s)	1.86	1.26	1.15	2.56	67
sGaw (1/cmH2O*s)	0.2	0.39	0.14	0.26	191	sGaw (1/cmH2O*s)	0.2	0.31	0.14	0.26	157

## Discussion

Observations

IPF management typically revolves around the early initiation of antifibrotic agents, pirfenidone and nintedanib [1-2,6-7]. Patients who are recalcitrant to this standard approach are considered for lung transplantation [1,6]. However, patients placed on a transplant waiting list often perish prior to their transplant, as wait times may be up to two to three years [1]. Our patient forewent this intervention and instead pursued adjunctive alternative treatments, as offered in this case report.

Lessons

While this is not the first study to demonstrate the positive effects adjunctive or alternative treatments have on patients with IPF, it shows how these manual therapy techniques can greatly enhance care for IPF patients. Vainshelboim reports that exercise training demonstrates short-term improvement in dyspnea, exercise capacity, and quality of life [8]. Additionally, studies have observed improvements in exercise capacity, quality of life, and functional capacities in patients who participated in pulmonary rehabilitation programs [9-12]. Cheng et al. reports in their analysis that pulmonary rehabilitation improves short-term, but not long-term, impacts on exercise capacity and health-related quality of life [13]. Further investigation is needed to elucidate the long-term impacts of pulmonary rehabilitation on IPF [13]. Other lung diseases such as chronic obstructive pulmonary disease (COPD), pneumonia, and tuberculosis have been shown to positively benefit from pulmonary rehabilitation as a treatment [14-16].

Respecting the restrictive nature of this disease, the rehabilitation within this case prioritized increased mobility of the respiratory system. Joint mobilization with an activator focused on aligning the joints and increasing mobility of the spine, thus allowing for improved ventilatory function. Trunk rotation with arm elevation and intercostal muscle stretching with MRT directly lessened the anatomic restrictions developed around the respiratory system, thus allowing for full expansion and contraction of the lungs. Additionally, the assignment of at-home self-led therapy recruited the patient to adopt a responsible change-driven mindset. The culmination of these techniques was shown to be positive, with a decrease in symptom burden, improved respiratory function, and oxygenation.

## Conclusions

This patient's positive response to a non-invasive technique after failing the standard of care demonstrates a benefit to the consideration and utilization of alternative treatments, such as manual therapy. These positive responses were observable in different aspects of her life, including an improved lifestyle and ability to complete activities she was previously unable to do. Additionally, her PFT values stabilized, and her oxygen saturation improved. Further research is needed to evaluate the effect of this treatment protocol on patients of different ages and backgrounds. The non-invasive nature of manual therapy and positive response in this patient support further exploration and supplementation in clinicians' knowledge of treatment options for those who have failed the standard of care. 
